# Expansion of donor-unrestricted MAIT cells with enhanced cytolytic function suitable for TCR redirection

**DOI:** 10.1172/jci.insight.140074

**Published:** 2021-03-08

**Authors:** Tiphaine Parrot, Katie Healy, Caroline Boulouis, Michał J. Sobkowiak, Edwin Leeansyah, Soo Aleman, Antonio Bertoletti, Margaret Sällberg Chen, Johan K. Sandberg

**Affiliations:** 1 Center for Infection Medicine, Department of Medicine, and; 2 Division of Clinical Diagnostics and Surgery, Department of Dental Medicine, Karolinska Institutet, Stockholm, Sweden.; 3 Tsinghua-Berkeley Shenzhen Institute, Tsinghua University, Shenzhen, China.; 4 Program in Emerging Infectious Diseases, Duke-National University of Singapore Medical School, Singapore.; 5 Department of Infectious Diseases, Karolinska University Hospital, Stockholm, Sweden.

**Keywords:** Immunology, Cellular immune response, Immunotherapy, T cells

## Abstract

Progress in our understanding of MR1-restricted mucosa-associated invariant T (MAIT) cells has raised interest in harnessing these cells for immunotherapy. The innate-like response characteristics, abundance in the blood, donor-unrestricted nature, and tropism for tissues make MAIT cells suitable candidates for adoptive cell transfer therapies. However, reliable methods and tools to utilize MAIT cells in such approaches are lacking. Here, we established methodology for efficient expansion of human MAIT cells in culture with high purity and yield, while preserving their functional response toward their natural ligand and increasing their cytotoxic potential. The cultured MAIT cells retained their effector memory characteristics without signs of terminal differentiation and expressed a more diverse set of chemokine receptors, potentially widening their already broad tissue tropism. To investigate the potential of MAIT cells in a context outside their main role in controlling bacterial infection, we engineered cultured MAIT cells with a new TCR specificity to mediate effective antiviral HLA class I–restricted effector function. In summary, we developed robust and effective methodology for the expansion of human MAIT cells with enhanced cytolytic capacity and for their engineering with a new specificity. These findings form a basis for the development of MAIT cells as a platform for adoptive immunotherapy.

## Graphical abstract

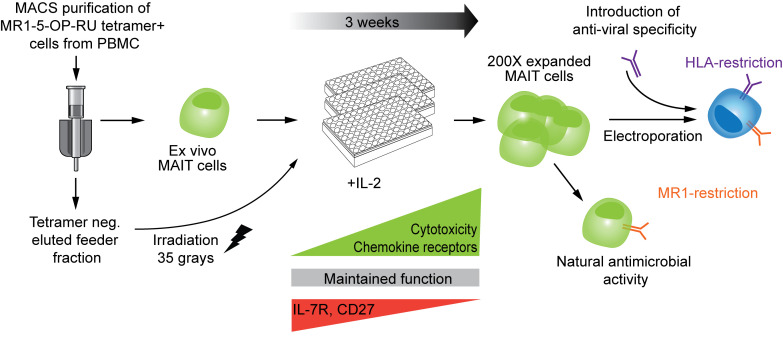

## Introduction

Mucosa-associated invariant T (MAIT) cells are a nonconventional T cell population at the bridge between innate and adaptive immunity (1, 2). Human MAIT cells express a semi-invariant TCR using the Vα7.2 (TRAV1-2) TCR segment paired with a restricted TCRβ repertoire, which specifically recognizes vitamin B2–derived microbial metabolites loaded on the highly conserved MR1 molecule (3–5). This limited TCR repertoire allows for the identification of MAIT cells based on the coexpression of Vα7.2 and CD161 (6) or, more recently, using MR1 tetramers loaded with the stimulatory MAIT cell ligand 5-(2-oxopropylideneamino)-6-D-ribitylaminouracil (5-OP-RU) (7, 8).

Abundant in the blood, where they account for 1%–10% of total T cells (9, 10), MAIT cells are enriched in mucosal tissues as well as in peripheral organs, including the liver, where they represent up to 30%–50% of T cells (11). The metabolites recognized by MAIT cells are byproducts of the riboflavin biosynthesis pathway expressed by diverse species of bacteria, mycobacteria, and fungi (12); therefore, in association with their location at sites of pathogen entry, MAIT cells are viewed as early sentinels responding to microbial infections. In response to antigen, MAIT cells produce proinflammatory cytokines, such as IFN-γ, TNF, and IL-17A, and can release cytolytic effector molecules, including granzymes and perforin, leading to the lysis of the infected cells, the inhibition of bacterial growth, and the shaping of the local immune response (9, 13–15). MAIT cells can also be activated in a TCR- and MR1-independent way via the proinflammatory cytokines IL-12 and IL-18, triggering mainly IFN-γ production (16–18). This innate property broadens the MAIT cell response beyond bacteria to play a role also in response to viruses and in inflammatory disorders (19–22).

Several studies reported a protective role of MAIT cells in bacterial and viral infections in murine models, supporting the potential utility of MAIT cells in a therapeutic setting (23–27). The use of MAIT cell ligands during infection or before infection as an adjuvant for vaccination or as a prophylactic treatment was proposed as an option to boost the MAIT cell response (23, 26). In addition, the potential application of MAIT cells in regenerative medicine or as a new immunotherapeutic approach in cancer, such as CAR MAIT cells, has been proposed (1, 28).

MAIT cells have several features that make them an attractive candidate for adoptive cell transfer therapies (28, 29). These include a semi-invariant TCR repertoire restricted to the highly conserved and nonpolymorphic MR1 molecule. Surface expression of MR1 is tightly regulated and very low in the absence of riboflavin metabolite antigen (30), thus reducing the risk for off-target side effects. MAIT cells are abundant in blood of humans, and therefore, a good number of cells can be isolated from a limited volume of blood. Furthermore, MAIT cells are a homogenous population, with an effector memory–like phenotype (9, 18), capable of responding fast without the need for priming. Finally, their natural tropism for tissues, such as liver and lung (9), could potentially be harnessed to target conditions in tissues that require tissue-infiltrating properties.

To evaluate the therapeutic potential, safety, and possible applications of adoptively transferred MAIT cells, there is a need to develop effective ex vivo expansion methods combining consistent expansion in culture with conserved features. So far, the main known strategy for the generation of large numbers of MAIT cells is through the generation of iPC-derived MAIT cells, a technically challenging approach (31). In the present study, we optimized methodology for ex vivo expansion of high numbers of MAIT cells isolated from human peripheral blood. We show that the expanded MAIT cells retain their overall functional properties, with a notable enhancement of their cytolytic capacity. Furthermore, the expanded MAIT cells display limited expression of checkpoint inhibition receptors and a broadened tissue-homing chemokine receptor repertoire. Finally, we show that these cells can be engineered to express a new functional TCR, endowing these cells with antiviral specificity. These methods and findings provide a platform for the evaluation of human MAIT cells in immunotherapy.

## Results

### Effective ex vivo expansion of magnetic bead–sorted MAIT cells.

**Figure 1:**
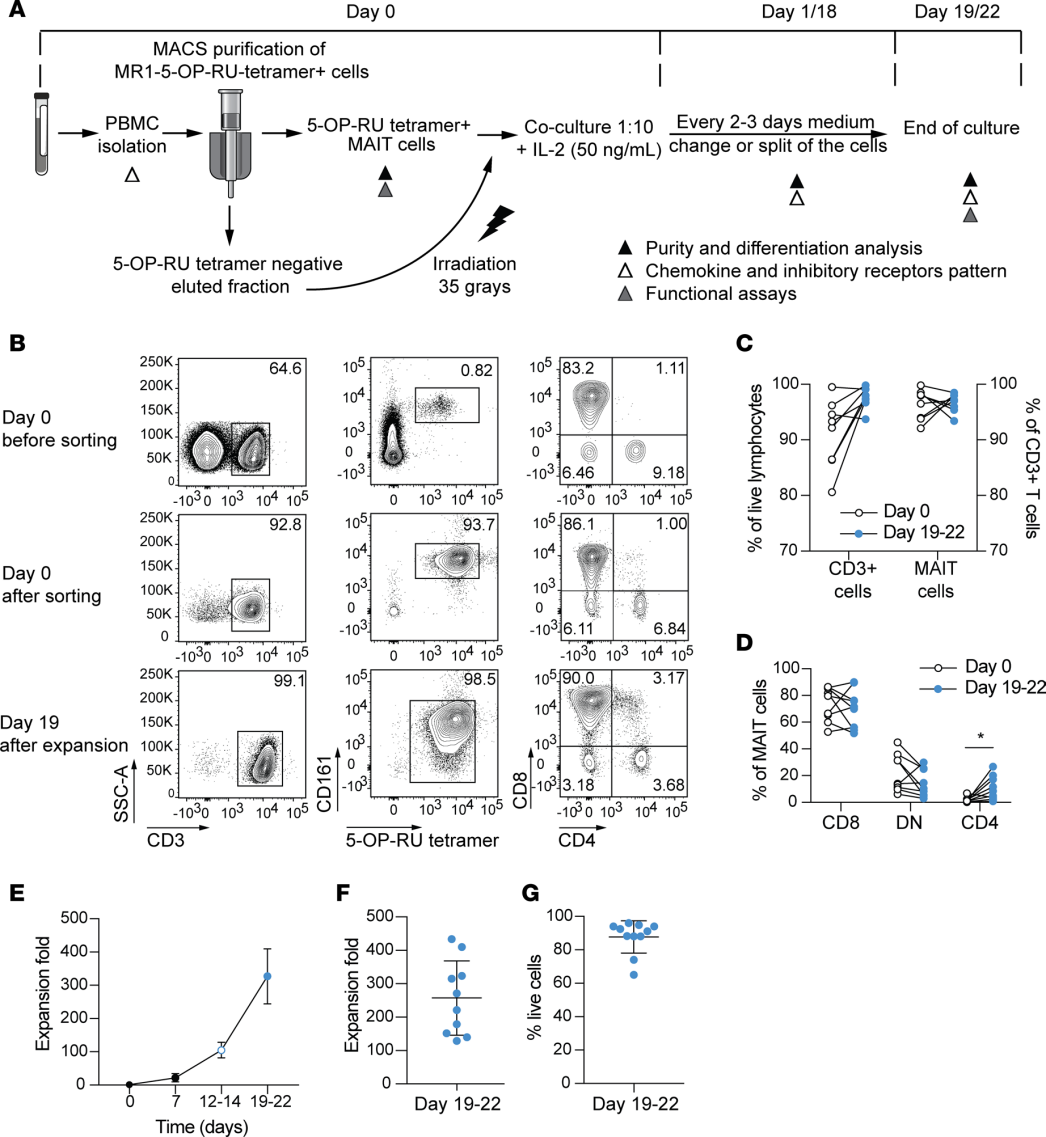
Qualitative validation of the optimized expansion protocol for MAIT cells. (**A**) Schematic representation of the developed expansion method protocol for MAIT cells. (**B**) Representative staining of MAIT cell purity and subset distribution before and after immunomagnetic enrichment and following 3 weeks of expansion culture. (**C**) Percentages of total CD3^+^ cells of live cells and percentages of total MAIT cells of CD3^+^ cells identified at day 0 (white circles) and at days 19 to 22 (blue circles) of culture (*n* = 9). (**D**) Percentages of CD8^+^, CD4^+^, and DN MAIT cell subsets before (white circles) and after 3 weeks of expansion culture (blue circles) (*n* = 9). (**E**) Monitoring of the expansion fold of MAIT cells over time. The expansion fold was defined as the ratio between the number of MAIT cells inoculated at day 0 and the number of MAIT cells obtained at the end of the expansion culture, as determined by cell counting and flow cytometry (*n* = 4). (**F**) Expansion fold and (**G**) viability of MAIT cells after 3 weeks of expansion culture (*n* = 9 and *n* = 10, respectively). (**C** and **D**) The Wilcoxon’s signed rank test was used to detect significant differences between paired groups. *P < 0.05. (**E–G**) Graphs represent mean ± SD.

MAIT cells represent a large population in the peripheral blood of healthy humans. To evaluate the possibility of using this consistent source of cells for direct expansion ex vivo, MAIT cells were enriched using immunomagnetic bead enrichment based on positive staining with the MR1-5-OP-RU tetramer (Supplemental Figure 1A; supplemental material available online with this article; https://doi.org/10.1172/jci.insight.140074DS1). We next evaluated different culture conditions to support optimal MAIT cell expansion, combining efficient expansion with high purity while using a restricted number of exogenous reagents. The efficacy of different concentrations of cytokines, including IL-2, IL-15, and IL-7, was evaluated alone or in combination with nonspecific activation using anti-CD3/CD2/CD28 soluble tetrameric complexes, and/or irradiated autologous PBMCs or monocytes as feeder cells, in serum-free media or media supplemented with CTS Immune Cell Serum Replacement (Supplemental Figure 1B). This sequential optimization approach suggested that IL-7 was not optimal for MAIT cell expansion in vitro, since when used alone or in association with anti-CD3/CD2/CD28 stimulation or feeder cells, IL-7 supported poor proliferation with an expansion fold (EF) below 5 irrespective of the concentration used (1–100 ng/mL tested). On the other hand, both IL-2 and IL-15 alone were able to promote a slight proliferation of MAIT cells (EF up to 10), possibly in synergy with a weak activation signal provided by the MR1 tetramer bound to immunomagnetic beads. Addition of anti-CD3/CD2/CD28 stimulation did not enhance the proliferation over that seen with IL-2 or IL-15 alone (EF up to 12). However, the use of irradiated autologous PBMCs as feeder cells strongly promoted MAIT cell expansion without loss of purity with an optimal effect at a 1:10 ratio. This proliferation was further improved in media supplemented with CTS Immune Cell Serum Replacement. Addition of anti-CD3/2/28 stimulation to feeder cells supported greater global EF, but this resulted in the proliferation of contaminating T cells reducing the MAIT cell purity below 40%. Overall, both IL-2 and IL-15 supported similar EF, but because IL-2 gave more reproducible results, we decided to use this cytokine in the further experiments. Overall, culture of MR1-5-OP-RU tetramer–sorted MAIT cells with autologous PBMCs at a 1:10 ratio, in the presence of IL-2 at 50 ng/mL in 8% serum replacement supplemented media, was the most optimal of conditions tested, leading to high MAIT cell expansion and purity (Figure 1A).

### Qualitative characterization of MAIT cell cultures.

After immunomagnetic bead isolation with the MR1 tetramer, MAIT cells represented 96% of CD3^+^ cells on average (Figure 1, B and C). Rare contaminating non–T cells did not grow and were lost during cell culture, resulting in CD3^+^ enrichment at the end of the expansion (ranging from 91% to 98%). After 3 weeks of culture, the purity of MAIT cells was stable at above 95%, as assessed by tetramer staining (Figure 1, B and C). It should be noted that, compared with ex vivo MAIT cells, the expanded MAIT cells expressed a heterogeneous dim or low level of CD161. Compared with ex vivo MAIT cells, the expanded MAIT cells were similarly majority CD8^+^ but showed a decreased frequency of CD4 CD8 double-negative (DN) cells and a slightly larger CD4^+^ fraction (Figure 1D). Monitoring of MAIT cell expansion cultures over time showed that cells started to proliferate after 7 days and grew exponentially for 2 more weeks (Figure 1E). After 3 weeks of expansion, cultures reached an average EF of 258 (Figure 1F), ranging from 100 to 400 and with a viability above 85% (Figure 1G). Combining this EF with the abundance of MAIT cells in peripheral blood of healthy donors, we estimate that up to 1.9 × 10^9^ MAIT cells can be generated on average from 50 mL buffy coat from healthy donors (Supplemental Table 1). In patients with chronic illnesses, the frequency and functional properties of peripheral blood MAIT cells can be severely affected, possibly limiting their expansion. To address this concern, we applied our expansion protocol to peripheral blood MAIT cells derived from chronically HBV-infected individuals described to have a reduced MAIT cell frequency (32, 33). These patient-derived MAIT cells expanded well under these conditions to an extent similar, to that in healthy donors (Supplemental Figure 1C). The use of IL-15 or allogeneic PBMCs as feeder cells did not further improve the expansion. In conclusion, we developed a robust and effective strategy to generate high numbers of MAIT cells at high purity from healthy donors as well as patients with viral hepatitis.

### Expansion cultured MAIT cells retain their functional response toward bacterial and cytokine stimulation and are pre-armed for cytolysis.

**Figure 2:**
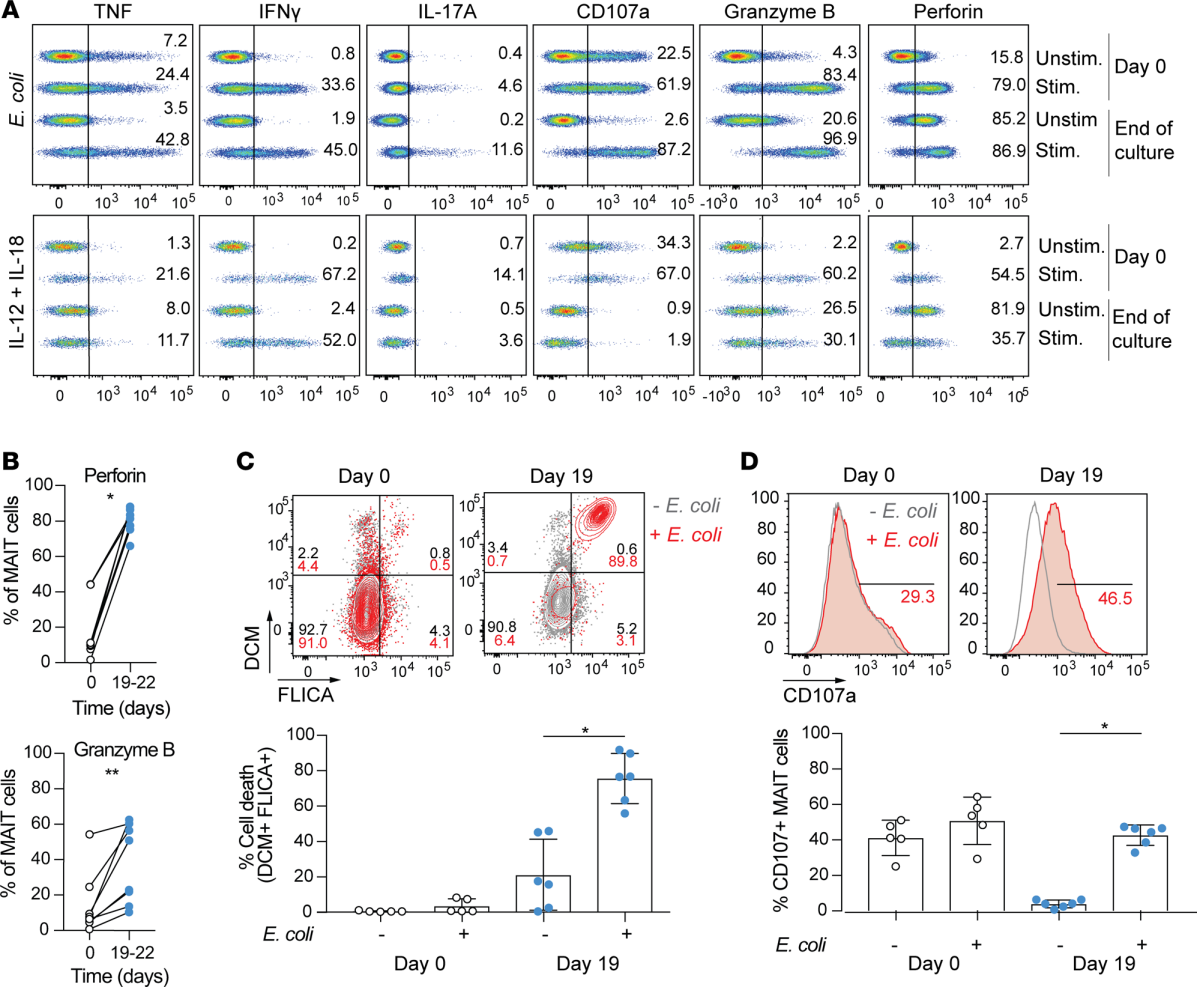
Expanded MAIT cells equally respond to bacterial and cytokine stimulation but have a more potent cytolytic potential than ex vivo MAIT cells. (**A**) Representative expression of TNF, IFN-γ, IL-17A, CD107a, granzyme B, and perforin by MAIT cells at baseline and upon *E*. *coli* and IL-12/IL-18 stimulation for 24 hours before (day 0) and after (day 19) expansion culture. (**B**) Percentages of expression of perforin and granzyme B in MAIT cells at rest and before (day 0) and after (day 19–22) expansion (*n* = 7 and *n* = 8, respectively). Representative examples and average frequency of (**C**) cell death in 293T-hMR1 cells and of (**D**) CD107a expression on MAIT cells following coculture for 24 hours of 293T-hMR1 cells with ex vivo (day 0, white circles) or expanded (day 19, blue circles) MAIT cells in presence or absence of *E*. *coli* (*n* = 5–6). 293T-hMR1 cell death was defined as cells double positive for dead cell marker (DCM^+^) and activated caspases, a marker of apoptosis (FLICA^+^). (**B–D**) The Wilcoxon’s signed rank test was used to detect significant differences between paired groups. *P < 0.05, **P < 0.01. (**C** and **D**) Graphs represent mean ± SD.

Next, we aimed to characterize the functional integrity and profile of the cultured MAIT cells after expansion (expansion cultured MAIT cells) in comparison to their ex vivo counterparts. MAIT cell cytokine expression after isolation at day 0, and after 3 weeks of expansion was compared after stimulation with THP-1 cells fed mildly fixed *E*. *coli*, or after IL-12 and IL-18 stimulation, for 24 hours (Figure 2A). Following bacterial stimulation, ex vivo MAIT cells produced high levels of the proinflammatory cytokines TNF and IFN-γ, upregulated their cytolytic granule granzyme B and perforin content, and were able to degranulate, whereas their expression of IL-17A was low. This response and expression pattern were largely retained by expanded MAIT cells (Figure 2A and Supplemental Figure 2A). We next compared the level of MR1 dependency of MAIT cell responses before and after expansion. Addition of MR1 blocking antibody abrogated the responses of ex vivo and expanded MAIT cells to a similar extent (Supplemental Figure 2B). Although not statistically significant, the induction of granzyme B and perforin by expanded cells seemed less MR1 dependent than before expansion.

In response to IL-12 and IL-18 stimulation, both the ex vivo and expanded MAIT cells similarly expressed high levels of IFN-γ, perforin, and granzyme B, while they expressed low to no expression of TNF and IL-17A (Figure 2A and Supplemental Figure 2C). The minor difference detected was in the surface expression of CD107a observed at day 0, despite degranulation being primarily TCR dependent (Supplemental Figure 2B). One may speculate that this might be the result of TCR activation of ex vivo MAIT cells via cross-linking by the MR1 tetramer. The expanded MAIT cells displayed an increased basal level of granzyme B and perforin (Figure 2, A and B). We hypothesized that this elevated basal cytolytic granule content might be associated with an enhanced cytotoxic effector function. Indeed, the 3-week cultured MAIT cells were able to efficiently kill *E*. *coli*–fed 293T-hMR1 cells, whereas fresh ex vivo MAIT cells were not (Figure 2C), despite similar ability to degranulate (Figure 2D). We next investigated whether this cytolytic pre-arming of expanded MAIT cells may lead to TCR-independent “bystander” killing of cells. Similar to the ex vivo MAIT cells, degranulation and killing activity of expanded MAIT cells in response to *E*. *coli*–fed 293T-hMR1 cells was MR1 dependent and was not triggered by stimulation with proinflammatory cytokines alone (Supplemental Figure 2D). Furthermore, the expanded MAIT cells did not develop NK cell–like cytolytic activity against K562 target cells (Supplemental Figure 2E), as was previously described to occur in liver MAIT cells stimulated with IL-15 (34). Therefore, in addition to their normal functional response pattern, expanded MAIT cells are pre-armed as cytolytic effectors in response to TCR-mediated recognition of antigen.

### Expansion cultured MAIT cells retain their effector memory phenotype without signs of exhaustion or senescence.

**Figure 3:**
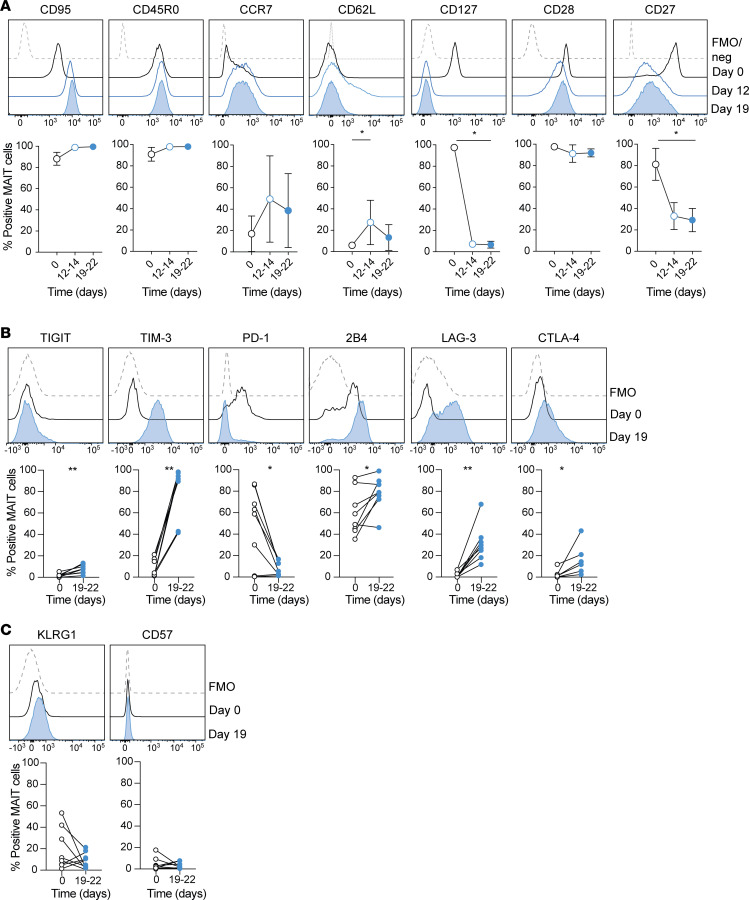
Expanded MAIT cells show signs of activation and advanced maturation rather than exhaustion and terminal differentiation. Representative example and average expression of the indicated (**A**) maturation markers, (**B**) inhibitory receptors, and (**C**) senescence markers on MAIT cells over time in culture (*n* = 6–8). (**A**) Graphs represent the mean ± SD. (**A**) The nonparametric Friedman’s test and (**B** and **C**) the Wilcoxon’s signed rank test were performed to detect significant differences between paired groups. *P < 0.05, **P < 0.01.

With their effector memory phenotype, including expression of CD95, CD45RO, CD28, CD27, and CD127, paired with low CCR7 and CD62L expression, MAIT cells have the capacity to home to peripheral tissue, to persist without antigen stimulation, and to launch a rapid and strong response following activation. There is a risk that long-term in vitro expansion may alter the status of T cells toward a more differentiated signature, reducing their persistence and efficacy for clinical application (35). Therefore, we investigated if MAIT cell differentiation status was compromised over the course of the 3-week expansion culture. MAIT cells retained their expression pattern of CD95, CD45RO, and CD28. However, a reduced expression of CD27 and almost complete loss of CD127 expression was evident already half-way through the culture (Figure 3A). Withdrawal of IL-2 during an additional 7-day culture partially restored CD127 expression, whereas CD27 expression was unchanged (Supplemental Figure 3A). Interestingly, transient upregulation of both CCR7 and CD62L occurred during the first half of expansion, receding to the baseline level at the end of culture.

Since T cell differentiation and activation status are associated with the acquisition of inhibitory receptors that modulate the T cell response, we next evaluated the expression of a panel of such receptors (Figure 3B and Supplemental Figure 3B). At baseline, ex vivo MAIT cells expressed intermediate and variable levels of PD-1 and 2B4, while they were positive for CD96. Following expansion culture, the expression of PD-1 was lost while the expression of 2B4 was increased, and expression of LAG-3 and CTLA-4 was induced to moderate levels. TIM-3 was consistently expressed at relatively high levels in the expanded MAIT cells, reflecting their activation. TIM-3 expression progressively decreased as MAIT cell proliferation slowed down and decreased even more after withdrawal of IL-2 (Supplemental Figure 3A). Because TIM-3 and LAG-3 were induced during culture, one might speculate that these receptors could negatively affect MAIT cell responses toward *E*. *coli*. However, there was no detectable change in MAIT cell response when blocking antibodies for TIM-3 and LAG-3 were added during the stimulation of MAIT cells with *E*. *coli*–fed THP-1 cells (Supplemental Figure 4A). In a slightly different experimental approach, the cross-linking of TIM-3 and LAG-3 on MAIT cells using specific monoclonal antibody–coated P815 cells had no clear effect on MAIT cell cytokine expression (Supplemental Figure 4B). Thus, under these experimental settings, the induced expression of TIM-3 and LAG-3 on MAIT cells had no detectable inhibitory effect on MAIT cell activation.

Finally, in line with a relatively mature, but nonterminally differentiated phenotype, the expanded MAIT cells did not express CD57 and KLRG1 (Figure 3C). Overall, expanded MAIT cells retain nonexhausted effector memory–like characteristics, with some signs of advanced differentiation characterized by the reversible loss of CD127 and reduced expression of CD27.

### Broadened chemokine receptor expression pattern in expansion cultured MAIT cells.

**Figure 4:**
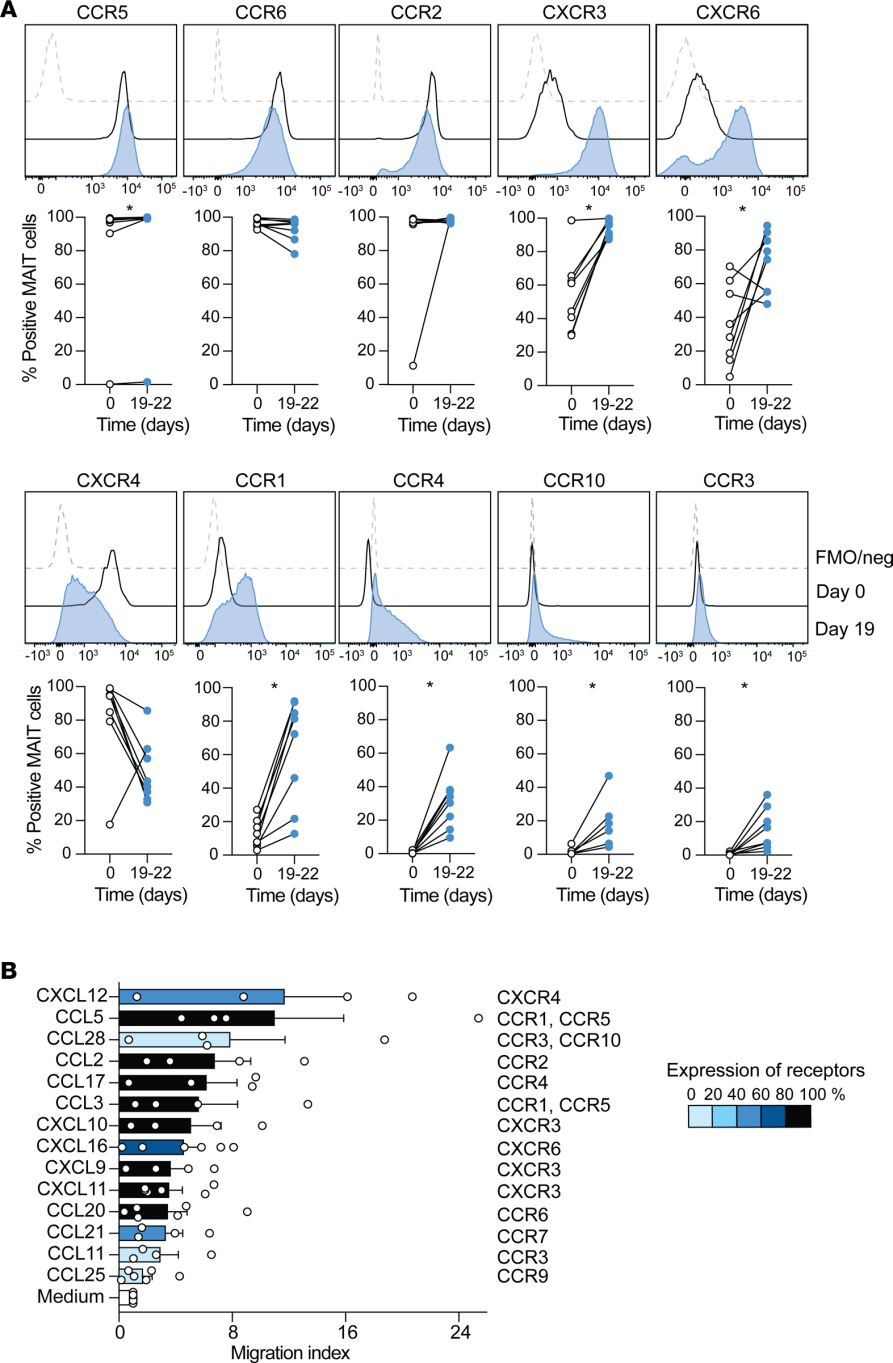
Expanded MAIT cells express a more diverse set of functional chemokine receptors than ex vivo MAIT cells. (**A**) Representative and average expression of the indicated chemokine receptor on ex vivo (day 0) and expanded (day 19–22) MAIT cells (*n* = 6). (**B**) Migration index of expanded MAIT cells toward the indicated chemokine. The migration index was defined as the ratio between the absolute number of MAIT cells collected in the bottom chamber in the presence of chemokine and the number of MAIT cells collected in the bottom chamber in the presence of control media.

Human MAIT cells are known to express several chemokine receptors, allowing their recruitment to peripheral tissues (9). Consistent with previous observations, ex vivo MAIT cells expressed high levels of CCR5, CCR6, CCR2, and CXCR4 and intermediate variable levels of CXCR3 and CXCR6 (Figure 4A) (9). After the 3-week expansion culture, CCR5, CCR6 and CCR2 expression remained highly expressed, while CXCR3 and CXCR6 were further induced and CXCR4 was downregulated (Figure 4A). Expansion cultured MAIT cells also expressed a new set of chemokine receptors, including CCR1 and CCR4, and low but detectable levels of CCR10 and CCR3, which were not expressed at detectable levels ex vivo. No expression of CXCR5, CXCR1, CX3CR1, CXCR2, CCR9, or CXCR7 was detected either before or after culture (Supplemental Figure 3C). We next evaluated if the expressed chemokine receptors were functional and sufficient to trigger MAIT cell migration toward the associated chemokines. Trans-well assay experiments confirmed the capacity of expanded MAIT cells to migrate in response to a panel of chemokines, in particular to CCL5 and CXCL12 (Figure 4B). Thus, MAIT cells diversify their chemokine receptor expression pattern during expansion culture.

### Engineering of MAIT cells toward an antiviral specificity.

**Figure 5:**
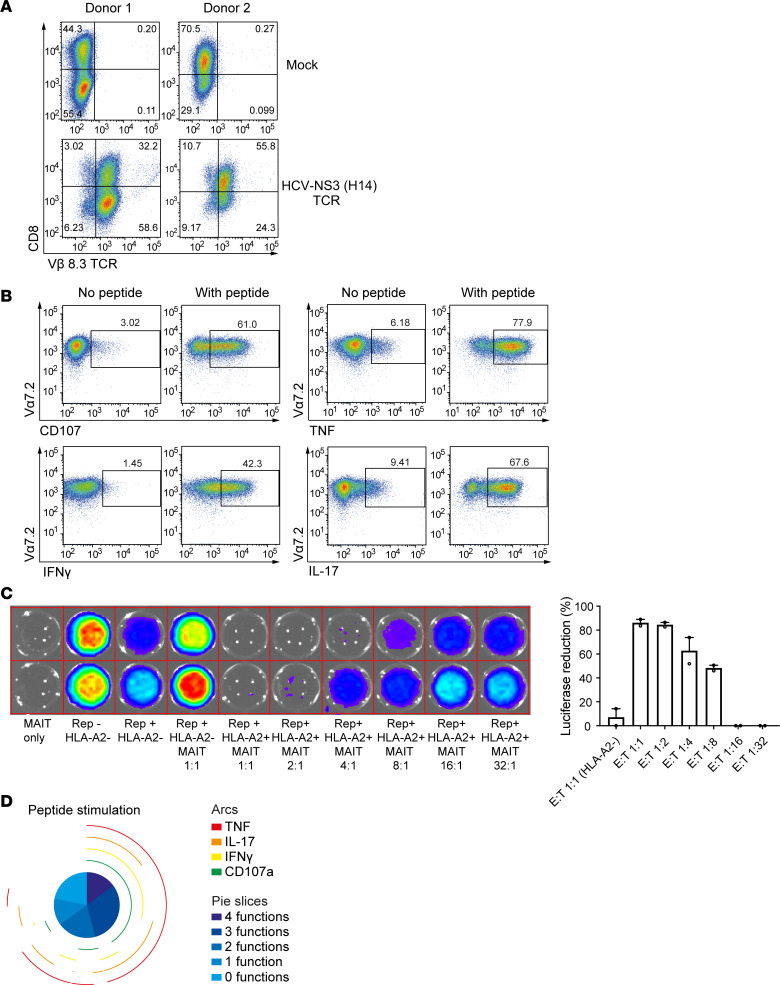
TCR transfection of expanded MAIT cells endows them with a new antiviral specificity. (**A**) HCV-specific TCR H4 (Vβ8.3) expression in CD8^+^ MAIT cells in 2 blood donors 18 hours following electroporation with or without mRNA encoding the TCR. (**B**) Representative FACS plot of cytokine responses of TCR-redirected MAIT cells following exposure to T2 target cells loaded with or without HCV NS3 1073 peptide. (**C**) TCR-transfected MAIT cell specific activity against HLA-A2^+^ Huh-7 HCVRep^+^ human hepatoma cells persistently replicating the HCV RNA replicon of genotype 1b Con1-ET assessed by suppression of luciferase activity (*n* = 2). (**D**) Pie charts describing the polyfunctionality and cytokine expression in HCV TCR–redirected MAIT cells in response to HCV NS3 peptide-pulsed T2 cells.

To evaluate whether MAIT cells cultured using our optimized methodology are suitable for TCR-redirected immunotherapeutic approaches, we next developed a protocol for mRNA transfection of expanded MAIT cells. Given the liver tropism of MAIT cells, we choose a hepatotropic pathogen hepatitis C virus (HCV) system, for which matching TCR and HCV RNA replicon (HCVRep^+^) human hepatoma target cells have been established (36). MAIT cells were expanded in culture as described above with consistent purity and quantity and transfected with mRNA encoding TCR H4, a mouse-derived Vβ8.3^+^ TCR specific for the HCV NS3-1073 HLA-A2–presented epitope. Surface expression of this TCR was confirmed using anti-mouse Vβ8.3 antibody staining (Figure 5A). The majority of MAIT cells expressed the H4 HCV TCR in both CD8^+^ and CD8^–^ MAIT cell subsets. Coculture of H4 HCV TCR–expressing MAIT cells with T2 target cells loaded with NS3-1073 peptide triggered peptide-specific activation of degranulation, as well as production of TNF, IFN-γ, and IL-17, assessed by intracellular cytokine flow cytometry (Figure 5B). H4 HCV TCR–expressing MAIT cells specifically recognized and eliminated HLA-A2^+^ Huh-7-Lunet HCVRep^+^ hepatoma cells persistently replicating the HCV RNA replicon of genotype 1b Con1-ET, as determined by suppression of luciferase activity in a dose-dependent and HLA-A2–dependent manner (Figure 5C) (36). Furthermore, the response of TCR H4–transfected MAIT cells was highly polyfunctional and specific for the NS3-1073 viral peptide (Figure 5D). These results confirm that TCR-redirected MAIT cells display a new antigen-specific response recognizing a viral target in an HLA-restricted and peptide-specific manner.

Finally, we asked whether the introduction of a new TCR would affect the ability of MAIT cells to respond via their endogenous TCR to MR1-restricted bacterial stimulation. The expression of cytokines and cytolytic effector molecules by TCR-transfected MAIT cells in response to *E*. *coli* stimulation was comparable to nontransfected or mock-transfected MAIT cells (Supplemental Figure 5). TCR-transfected MAIT cells stimulated with NS3-1073 HCV peptide expressed high levels of IL-17 (Figure 5B). However, in response to *E*. *coli* stimulation IL-17 production was limited and similar to that of unmodified MAIT cells (Supplemental Figure 5). This suggests that in this case the newly introduced TCR delivers a stronger activating signal, allowing enhanced IL-17 expression.

## Discussion

Unconventional subtypes of T cells have specialized functions in the immune system to broaden the repertoire of antigens recognized beyond the classical peptide fragments presented by polymorphic HLA class I and II molecules (1, 2). These include T cells recognizing antigens presented by HLA class I–related molecules, such as MAIT cells recognizing MR1-presented antigens. MAIT cells and other unconventional T cells have several features that have recently attracted attention from an immunotherapeutic perspective (27, 29, 37). Here, we developed methodology for efficient ex vivo expansion of human MAIT cells isolated from peripheral blood. We show that these cells retain their functional profile with enhanced cytolytic capacity, limited expression of checkpoint inhibition receptors, and a broadened tissue-homing chemokine receptor repertoire. Finally, we show that the expanded MAIT cells can be engineered to express a second functional TCR, endowing these cells with antiviral specificity. These methods and findings provide a platform for the evaluation of human MAIT cells in immunotherapy and for detailed studies of the role of MAIT cells in health and disease.

Several cytokines can support MAIT cell proliferation, including IL-15, IL-2, or IL-12 (13, 38). The IL-7Rα is highly expressed on MAIT cells, and IL-7 treatment was recently shown to promote reconstitution of the CD8^+^ MAIT cell population in vivo in HIV-1–infected patients on antiretroviral treatment (39). However, in the culture conditions used here, IL-7 did not support strong MAIT cell expansion in vitro. Instead, both IL-2 and IL-15 were more efficient and supported similar levels of expansion. The MAIT cell subset composition was slightly changed during culture with an inverse ratio of CD4^+^ over DN cells compared with the peripheral blood. CD4^+^ MAIT cells were previously found to express higher levels of CD25 (40), and this may explain their preferential expansion in conditions supported by IL-2. MAIT cell subsets are phenotypically and functionally distinct, and the exact role of CD4^+^ MAIT cells is currently unknown (41). While DN MAIT cells were described as less functional than CD8^+^ MAIT cells and biased toward a Th17 signature (42), the CD4^+^ MAIT cells were described to be even less efficient at secreting IFN-γ, and TNF and unable to degranulate or to upregulate granzyme B expression (40). Due to the strong CD8 downregulation after activation, we were unable to strictly compare the activation profile of each subtype after expansion. However, the CD4^+^ MAIT cells were responsive and able to degranulate. At rest, the cytotoxic activity of peripheral blood MAIT cells is limited, and activation by their target or arming in response to cytokine stimulation is needed to achieve full cytolytic potential (13, 14, 43). Both IL-7 and IL-15 were previously shown to trigger upregulation of perforin and granzyme B in MAIT cells in a MR1-independent manner (14, 15, 34). During expansion culture, the MAIT cells were similarly primed by IL-2 and acquired high levels of granzyme B and perforin and potent cytolytic capacity. While liver MAIT cells have been shown to exert an NKG2A-mediated and MR1-independent cytolytic activity following culture in IL-15 (34), the cytolytic activity of MAIT cells using our protocol remained MR1 dependent to an extent similar to ex vivo MAIT cells.

Successful adoptive cell transfer relies on the ability of cells to persist in the new host, and this will likely be linked to their differentiation status. Less mature subtypes of T cells were previously shown to be more able to persist and to generate potent in vivo responses (44, 45). Here, expansion cultured MAIT cells overall retained an effector memory phenotype with a gradual reduction of CD127 and CD27 over time, indicative of a progressively more differentiated phenotype. A similar phenotype was obtained when using IL-15 as a supporting cytokine and was also reported for redifferentiated MAIT (reMAIT) cells generated from iPSCs (31), suggesting a degree of autonomous maturation of MAIT cells. Upon withdrawal of IL-2, CD127 expression was partially restored, whereas that of CD27 was not. To what extent the lower levels of CD27 and CD127 may compromise the function and survival of adoptively transferred MAIT cells in vivo remains to be determined. In this context, it is interesting to note that the expanded MAIT cells showed enhanced expression of the inhibitory receptors TIM-3 and to some extent LAG-3. However, blockade or cross-linking of these 2 receptors had no detectable effect on MAIT cell cytokine responses in vitro, suggesting a limited role for these receptors in controlling MAIT cell effector functions. Furthermore, TIM-3 expression in the cultured MAIT cells appeared transient and was drastically reduced after IL-2 withdrawal.

MAIT cells are known to express a broad range of chemokine receptors, including CCR5, CCR6, CCR2, CXCR3, and CXCR4, which are important for their infiltration of tissues, such as lung, liver, intestine, and bone marrow. Interestingly, a similar tropism pattern could be reconstituted in reMAIT cells derived from iPSCs, suggesting an inherent migratory program of MAIT cells (31). In our study, the expression of the above-mentioned chemokine receptors was conserved in cultured MAIT cells, and in addition there was an increased expression of CXCR3 and CXCR6, suggesting that while retaining their initial tropism potential, the in vitro expanded MAIT cells might have increased homing capacity to the liver and intestine. Despite the significant decrease in CXCR4 expression after expansion, MAIT cells were still able to efficiently migrate toward CXCL12. Additionally, new chemokine receptors emerged, including CCR1, CCR4, and CCR10, that could potentially expand the tissue tropism to include the skin.

MAIT cells have a natural specificity for MR1-presented riboflavin-derived microbial metabolites, and perhaps the most direct application of MAIT cells for adoptive transfer would be for chronic infections, with bacteria retaining the riboflavin biosynthesis pathway. This approach has gained support from the recent findings in *Legionella* infection in mice, where adoptively transferred MAIT cells afforded protection (23). The innate-like properties of MAIT cells could expand their use to include targeting of viral infections, as a protective role of MAIT cells has been described in influenza virus infection in mice (25). In many contexts, including, but not limited to, viral or bacterial infections and hepatic or pulmonary diseases, the peripheral MAIT cell compartment is severely reduced and does not recover, despite successful treatment while the residual MAIT cells are functionally impaired (32, 46–49). Although the mechanisms behind MAIT cell depletion are not clearly understood, loss of MAIT cells was associated with disease severity in several conditions and may increase the susceptibility to microbial infections. In these situations, replenishment of the MAIT cell compartment by adoptive cell transfer could represent a therapeutic option. Furthermore, we here demonstrated the possibility to redirect the antibacterial properties of MAIT cells toward a new antigen. After expansion culture, MAIT cells are permissive to transfection and ectopic expression of a TCR successfully conferring antiviral specificity and cytolytic function. In this system, we used an HLA-A2–restricted TCR specific for an HCV peptide epitope as a proof of concept and showed that the TCR-transfected MAIT cells were responsive toward the viral antigen. This occurred without compromising the endogenous specificity of the MAIT cells, as they maintained their antibacterial responsiveness. This work extends the application range of MAIT cell functional properties beyond bacterial infection.

The optimized methodology for MAIT cell expansion we describe here starts with magnetic bead–based isolation using the MR1 tetramer, and subsequent culture with IL-2 and irradiated autologous PBMC feeder cells, without cognate antigen stimulus. This approach for isolating MAIT cells can be replaced with FACS sorting based on the coexpression of Vα7.2 and CD161. However, if this alternative approach was used, then additional anti-CD3/CD2/CD28 stimulus was required at the start of culture (data not shown). Other types of mitogenic stimuli, such as phytohemagglutinin, can also be used to stimulate MAIT cell cultures (50). Our finding that such strong stimulus was not needed when the magnetic bead–based isolation using the MR1 tetramer was used suggests that the MR1 tetramer, which is refolded with 5-OP-RU agonist antigen, provides a weak stimulus to initiate proliferation. When this stimulus is combined with IL-2 or IL-15, together with the costimulatory ligands delivered by the irradiated feeder cell layer, this efficiently promotes MAIT cell expansion. The MR1 5-OP-RU tetramer specifically binds and may trigger MAIT cells, thus limiting the emergence of contaminating non-MAIT T cells at the end of the culture. In contrast, the unspecific CD3/CD2/CD28 activation preferentially promoted the expansion of the few non-MAIT cells contained in the positive fraction after the immunomagnetic sorting. Nevertheless, this effect could be circumvented by increasing the purity using FACS sorting. The EF obtained with our method was linked with the abundance of MAIT cells in blood and allowed an estimated production of 1.9 billion MAIT cells on average starting from 50 mL buffy coat. Functional and numerical impairment of MAIT cells has been reported in several diseases, such as chronic HBV infection. Importantly, we validated here that MAIT cells derived from HBV-infected patients were expandable at a similar range to that seen from healthy blood donors.

In conclusion, this work describes robust methodology for the culture and expansion of pure MAIT cells with retained functional properties and enhanced cytolytic capacity that can be modified toward an alternative antigen specificity for possible therapeutic applications.

## Methods

### Blood donors and cell lines.

Peripheral blood was obtained from healthy donors recruited at the Blood Transfusion Clinic and from HBV-infected patients followed at the Department of Infectious Diseases, both at the Karolinska University Hospital in Huddinge, Sweden. The 293T-hMR1 cell line (gift from Ted Hansen, Washington University, St. Louis, Missouri, USA) and THP-1, K562, T2, and P815 cells lines (all 4 from ATCC) were cultured in RPMI1640 complete medium supplemented with 25 mM HEPES, 2 mM L-glutamine (GE Healthcare), 10% FBS (MilliporeSigma), 50 μg/mL gentamicin (Thermo Fisher Scientific), and 100 μg/mL Normocin (Invivogen). Hepatoma Huh-7-Lunet cells, designated as Lunet-HLA-A2^+^ Luc-ubi-neo Con1 or Lunet-HLA-A2- Luc-ubi-neo Con1 (36), were cultured in complete DMEM medium (MilliporeSigma) supplemented with 10% FBS, 100 U/ml Penicillin, 100 μg/ml Streptomycin (MilliporeSigma), and 0.5 μg/ml G148 (Thermo Fisher Scientific). The Lunet-HLA-A2^+^ Luc-ubi-neo Con1 was further selected for by culture in the presence of 1 μg/ml puromycin (MilliporeSigma).

### MAIT cell isolation and expansion.

PBMCs were isolated by Ficoll-Hypaque density gradient centrifugation. MAIT cells were sorted by immunomagnetic separation after staining with the PE- or APC-conjugated 5-OP-RU MR1-tetramer (NIH tetramer core facility), followed by MACS anti-PE or anti-APC microbead enrichment according to the manufacturer’s instructions (Miltenyi Biotec). The eluted fraction containing MAIT cell–depleted PBMCs was irradiated at 35 Gray and resuspended at 2 × 10^6^ cells/mL in ImmunoCult Human T cell Expansion Medium (STEMCELL) supplemented with 8% CTS Immune Cell Serum Replacement (Thermo Fisher Scientific), 50 ng/mL animal-free recombinant human IL-2 (Peprotech), 100 μg/mL Normocin (MilliporeSigma), and 100 U/mL Penicillin/Streptomycin (GE Healthcare), and 100 μL/well (2 × 10^5^ cells) were added in a 96-well round-bottom plate. MAIT cells were resuspended at 4 × 10^5^ cells/mL in the same medium, and 50 μL (2 × 10^4^ cells) were added on top of the irradiated PBMC layer. Every 2 to 3 days the medium was changed by removing half of the volume or the cells were split by transferring half of the volume in a new 96-well plate and complete with 2× cytokine fresh medium. For the optimization steps, human recombinant IL-7 or IL-15 (Peprotech) and ImmunoCult Human CD3/CD28 T Cell Activator TCA (STEMCELL) were tested. For the purification of monocytes from blood, the RosetteSep Human Monocyte Enrichment Cocktail (STEMCELL) was used according to the manufacturer’s instruction.

### Flow cytometry staining procedure and antibodies.

The following antibodies were used for flow cytometry staining. CD107a-BUV395 (clone H4A3), CD161-PeCy5 (clone DX12), CD69-BUV737 (clone FN50), TNF-PeCy7 (clone Mab11), granzyme B-AF700 (clone GB11), CD3-FITC (clone SK7), CCR5-BUV395 (clone 2D7), CCR10 BV421 (clone 1B5), CCR9-AF488 (clone 112509), CCR7-BUV395 (clone 150503), CXCR5-AF647 (clone RF8B2), CD3-AF700 (clone UCHT1) were from BD Biosciences. CD8-BV570 (clone RPA-T8), CD3-BV650 (clone OKT3), CD4-BV711 (clone OKT4), Vα7.2-PE (clone 3C10), IL-17A-BV421 (clone BC168), Perforin-FITC (clone D48), IFN-γ-BV785 (clone 4SB34), CX3CR1-BV421 (clone 2A9-1), CCR6-BV650 (clone G034E3), CXCR4-BV785 (clone 12G5), CXCR3-Pe-Cy7 (clone G025H7), CXCR6-AF647 (clone K041E5), CCR3-BV510 (clone 5E8), CD3-BV785 (clone OKT3), CXCR2-Pe-Cy7 (clone 5E8), CXCR1-APC (clone 8F1), CCR4-BV421 (clone L291H4), CCR2-BV510 (clone K036C2), CCR1-AF488 (clone 362905), CXCR7-Pe-Cy7 (clone 8F11-M16), CD127-BV421 (clone A019D5), CD45RO-BV570 (clone UCHL1), CD8-BV650 (clone RPA-T8), TIM-3-BV785 (clone F38-2E2), CD28-PeDazzle594 (clone CD28.2), CD27-Pe-Cy7 (clone M-T271), CD95-APC (clone DX2), CD62L APC-Fire (clone DREG-56), CD96-BV421 (clone NK92.39), KLRG1-BV785 (clone 2F1/KLRG1), CD57-FITC (clone BC96), BTLA-Pedazzle594 (clone MIH26), TIGIT-Pe-Cy7 (clone A15153G), PD-1-BV421 (clone EH12.2H7), 2B4-Pedazzle594 (clone C1.7), CTLA-4-Pe-Cy7 (clone BN13), and LAG-3-AF647 (clone 11C3C65) were from Biolegend. The LIVE/DEAD Fixable Aqua and Near-IR Dead Cell Stain Kits (Thermo Fischer Scientific) were used for the staining of dead cells. For extracellular staining, cells were stained for 20 minutes at 4°C in PBS 2 mM EDTA and 2% FBS, washed, and fixed 10 minutes at room temperature in 1× BD CellFIX buffer (BD Biosciences) before analysis. If an intracellular staining step was following, cells were instead fixed for 30 minutes at 4°C in BD Cytofix/Cytoperm Fixation/Permeabilization Kit (BD Biosciences) and then stained for 30 minutes at 4°C in 1× Perm/Wash buffer (BD Biosciences).

### MAIT cell activation assays.

The MAIT cell activation assay was performed as previously described (38), with some adjustments. Freshly MR1 tetramer-sorted MAIT cells or pre-expanded MAIT cells were cocultured at a 1:1 ratio with THP-1 cells fed fixed *E*. *coli* (strain D21) at a bacteria per cell ratio of 30 for 24 hours. *E*. *coli* was mildly fixed for 3 minutes in 1× BD CellFIX (BD) with vortexing for the first minute and last 30 seconds and then washed repeatedly with PBS prior feeding to THP-1 for 3 hours. As indicated, 20 μg/mL purified anti-human MR1 antibody (26.5, Biolegend) or corresponding IgG2a isotype control (MOPC-173, Biolegend) was added during the last hour. MAIT cells were added in the presence of anti-CD107a mAb antibody (H4A3, BD), and 1 hour later the anti-CD28 mAb (L293, BD) at 1.25 μg/mL was added. Cells were incubated for 24 hours, and monensin (Golgi Stop, BD) and brefeldin A (Golgi Plug, BD) were added for the last 6 hours before staining. For IL-12 and IL-18 stimulation, freshly MR1 tetramer-sorted MAIT cells or pre-expanded MAIT cells were incubated for 24 hours with 10 ng/mL IL-12p70 and 100 ng/mL IL-18 for 24 hours, and monensin and brefeldin A were added for the last 6 hours before staining. For the cytotoxicity assay, freshly MR1 tetramer-sorted MAIT cells or pre-expanded MAIT cells were cocultured with 293T-hMR1 cells in the presence of 10 ng/mL IL-12p70 and 100 ng/mL IL-18 or with fed fixed *E*. *coli* (bacteria per cell ratio of 3) at a 1:10 ratio for 24 hours (38). As indicated, purified anti-human MR1 antibody (26.5, Biolegend) or corresponding IgG2a isotype control (MOPC-173, Biolegend) were added during the last hour. After 3 hours, MAIT cells were added in the presence of CD107a and FLICA reagent (Vybrant FAM Poly Caspases Assay Kit, Thermo Fisher Scientific), according to the manufacturer’s instruction, and incubated for 24 hours before staining. For the evaluation of NK cell–like cytotoxic activity, expanded MAIT cells were cocultured for 24 hours at a 1:10 ratio with K562 cells in the presence of CD107a and FLICA reagent. For the P815 assay redirected stimulation assay, expanded MAIT cells were cocultured for 6 hours at a 1:1 ratio with P815 cells preincubated for 1 hour at room temperature with anti-CD3 antibody (OKT3, Biolegend) in the presence of the blocking anti-LAG-3 (17B4, Adipogen) or anti-TIM-3 (F38-2E2, Biolegend) antibodies. All these assays were run in complete medium.

### Migration assays.

Migration assays were conducted in 5 μm pore Transwell 96-well plates (Corning HTS Transwell 96 well permeable supports). Here, 5 × 10^5^ MAIT cells in 100 μL serum-free complete medium were seeded in the upper chamber of the Transwell. The bottom chamber was filled with 200 μL of recombinant human chemokines at 200 ng/mL (all from R&D systems) in serum-free complete medium. As positive and negative controls, complete medium or serum-free complete medium were used, respectively. After 3 hours at 37°C, cells in the bottom chamber were stained and counted using FACS counting beads (Countbright Absolute Beads for Flow Cytometry, Thermo Fisher Scientific) according to the manufacturer’s instructions. The migration index was the ratio of the number of cells that migrated in the presence of the chemokine to the number of cells that migrated in the presence of the serum-free medium.

### TCR transfection.

The mRNA for TCR H4 specific for the HCV NS3 1073-1081 epitope was transcribed in vitro using the mMessage mMachine T7 Ultra kit (Thermo Fisher Scientific) according to the manufacturer’s instructions. On day 19–21 of the MAIT cell expansion culture, 1 × 10^7^ MAIT cells were suspended in 100 μL supplemented SF Cell Line Nucleofector Solution (Lonza) with 20 μg mRNA encoding the TCR. The suspension was transferred to a Nucleocuvette vessel and electroporated in a 4D-Nucleofector X Unit (Lonza). Immediately after pulsing, 400 μL prewarmed ImmunoCult Human T cell Expansion Medium (STEMCELL) supplemented with 8% CTS Immune Cell Serum Replacement (Thermo Fisher Scientific), 50 ng/mL animal-free recombinant human IL-2 (Peprotech), 100 μg/mL Normocin (MilliporeSigma), and 100 U/mL Penicillin/Streptomycin (GE Healthcare) were added to the cuvette and incubated at 37°C for 5 minutes. The contents of the cuvette were further collected to a final volume of 3 mL using the same medium and incubated overnight. 18–24 hours after electroporation, MAIT cell TCR expression was quantified by flow cytometry using a FITC-labeled anti-mouse Vβ8.3 antibody (BD Biosciences).

### TCR-redirected MAIT cell functional assays.

To evaluate the functional profile of the engineered MAIT cells, HLA-A2^+^ T2 cells were pulsed with 1 μg/ml HCV NS3 1073 peptide (CINGVCWTV) for 1 hour and washed twice. TCR-redirected MAIT cells (0.1 × 10^6^) were cocultured with empty or peptide-loaded T2 cells at a 1:1 ratio for 6 hours in the presence of CD107a antibody, monensin (Golgi Stop, BD), and brefeldin A (Golgi Plug, BD). At the end of the culture, MAIT cells were stained for IL-17A, IFN-γ, and TNF, as described above. Analysis and graphical presentation of MAIT cell polyfunctional data were performed using SPICE version 5.1 (downloaded from http://exon.niaid.nih.gov) (51).

### Bioluminescence assay.

Huh-7-Lunet-HLA-A2^+^ Luc-ubi-neo Con1–expressing cells or control Lunet-HLA-A2- Luc-ubi-neo Con1–expressing cells were seeded at 1 × 10^5^ cells/well in a 96-well plate and cultured overnight in complete DMEM without selection. HCV NS3 TCR–redirected MAIT cells were added at indicated ratios and cocultured for 24 hours. Medium was replaced with ONE-Glo Luciferase assay substrate (Promega), and bioluminescence was quantified using Living Image Software version 4.2 on the IVIS Spectrum instrument (Caliper Life Sciences).

### Statistics.

Statistical analyses were performed using Prism software v.6 (GraphPad). Statistically significant differences between paired samples were determined using Wilcoxon’s signed-rank test. For matched longitudinal analysis, the nonparametric Friedman’s test was performed with Dunn’s multiple comparison test. Two-sided *P* values of less than 0.05 were considered significant.

### Study approval.

The study was approved by the Regional Ethics Review Board in Stockholm, Sweden. Written informed consent was obtained from all blood donors and patients included in the study in accordance with the Declaration of Helsinki.

## Author contributions

JKS, TP, KH, SA, MSC, AB, and EL designed the experiments. TP, KH, and CB performed the experiments. TP, KH, MJS, and JKS analyzed the data. JKS and MSC supervised the work. JKS and TP wrote the paper. All authors reviewed and approved the final manuscript.

## Supplementary Material

Supplemental data

## Version 1 02/09/2021

In-Press Preview

## Version 2 03/08/2021

Electronic publication
