# Supplementary material for: Expansion of donor-unrestricted MAIT cells with enhanced cytolytic function suitable for TCR redirection
Source: JCI Insight. 2021 Mar 8;6(5):e140074. doi: 10.1172/jci.insight.140074 (PMC8021122; doi:10.1172/jci.insight.140074)
Supplement: Supplemental data [file jciinsight-6-140074-s221.pdf]

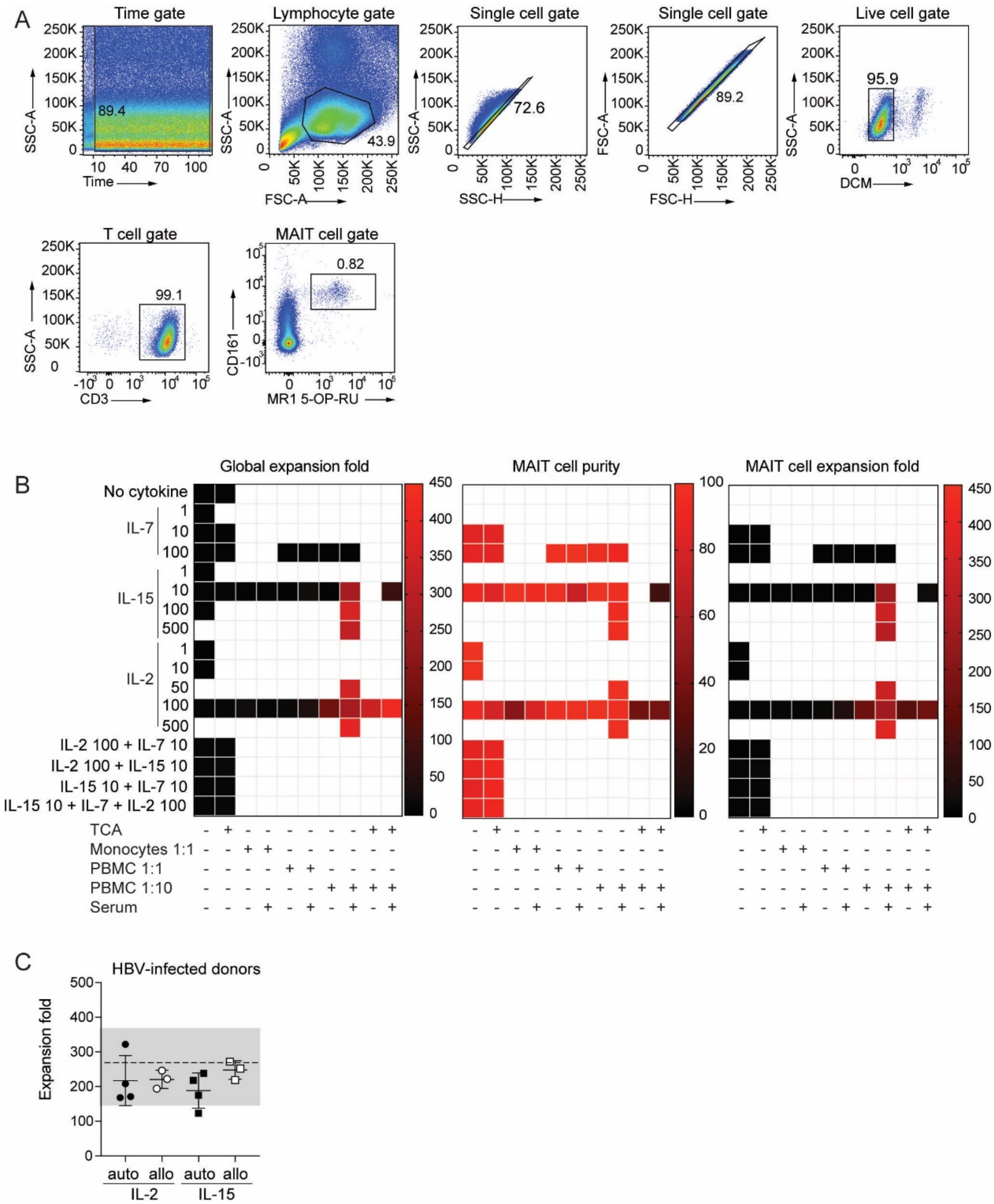

Supplemental Figure 1. Expansion fold and purity of MAIT cells following proliferation with diverse settings. (A) Gating strategy used to identify MAIT cells by flow cytometry for the determination of MAIT cell purity and functional response. For the chemokine receptor and inhibitory receptor phenotyping, MAIT cells were identified based on co-expression of Vα7.2 and CD161 at high levels. (B) Heat maps representing the total expansion fold, the purity and the expansion fold adjusted to MAIT cells reached after proliferation with different combinations of cytokines (row) and stimuli (column). The expansion fold was defined as the ratio between the total number of MAIT cells inoculated at day 0, and the total number of cells obtained after 3 weeks of expansion. The purity was defined by flow cytometry as the percentage of total MAIT cells in the CD3+ fraction. The expansion adjusted to MAIT cells was calculated as the multiplication of the two former parameters (n=3). (C) Expansion fold of MAIT cells from HBV-infected patients (mean ± SD) after 3 weeks of expansion culture in presence of 50 ng/mL IL-2 or 10 ng/mL IL-15 using autologous or allogenic PBMCs as feeder cells (n=3-4). Expansion fold of MAIT cells (mean ± SD) from healthy donors indicated in gray. Non-parametric Kruskal-Wallis and Dunn's post-hoc tests were used to detect significant differences between the groups.

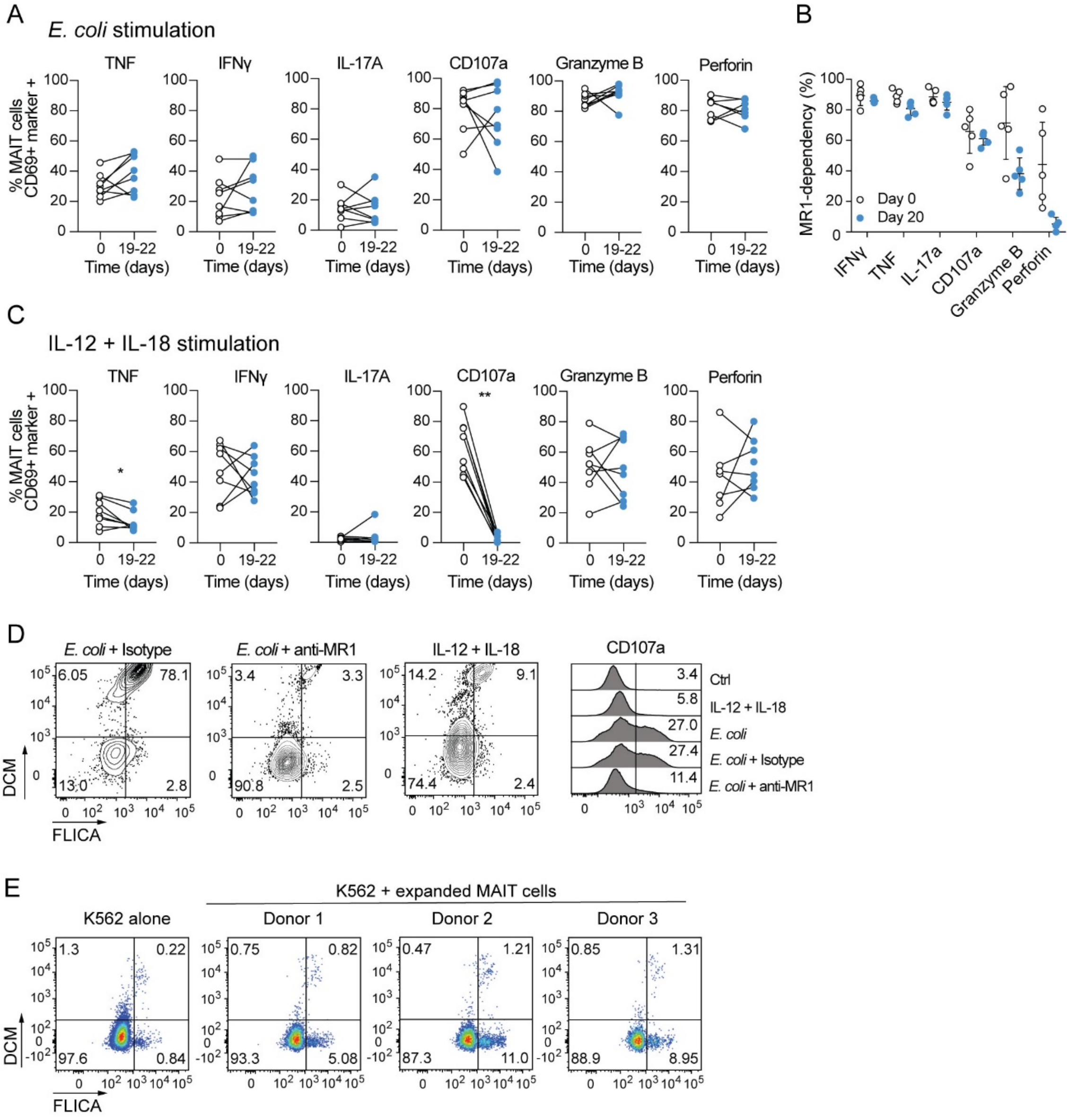

Supplemental Figure 2. Expression of cytokines and cytolytic effector molecules, and MR1-dependency of expanded MAIT cells. (A) Percentages of expression of TNF, IFNγ, IL-17A, CD107a, granzyme B and perforin in MAIT cells following stimulation with THP-1 cells fed mildly fixed *E. coli* before (day 0, black open circles) and after (day 19 to 22, open blue circles) expansion culture (n=7-8). (B) Percentage of MR1-dependency of MAIT cell response before and after expansion. Percentage of MR1-dependency was calculated as follows: ((% expression marker X in the isotype control condition - % expression marker X in the MR1 blocking condition) / (% expression marker X in the isotype control condition)) * 100. (C) Percentages of expression of TNF, IFNγ, IL-17A, CD107a, granzyme B and perforin in MAIT cells following stimulation with IL-12/IL-18 for 24 h before (day 0, black open circles) and after (day 19 to 22, open blue circles) expansion culture (n=7-8). (D) Expanded MAIT cell killing of 293T-MR1 cells in the presence of *E. coli*, *E. coli*+anti-MR1, or IL-12 and IL-18. Right panel: CD107a expression on expanded MAIT cells following coculture in the aforementioned culture conditions. (E) Representative examples of cell death (defined as DCM+ and FLICA+ cells) in K562 cells following co-culture for 24 h with expanded MAIT cells. In (A, B) the Wilcoxon's signed rank test was used for statistical analysis of paired data. *p < 0.05, **p < 0.01.

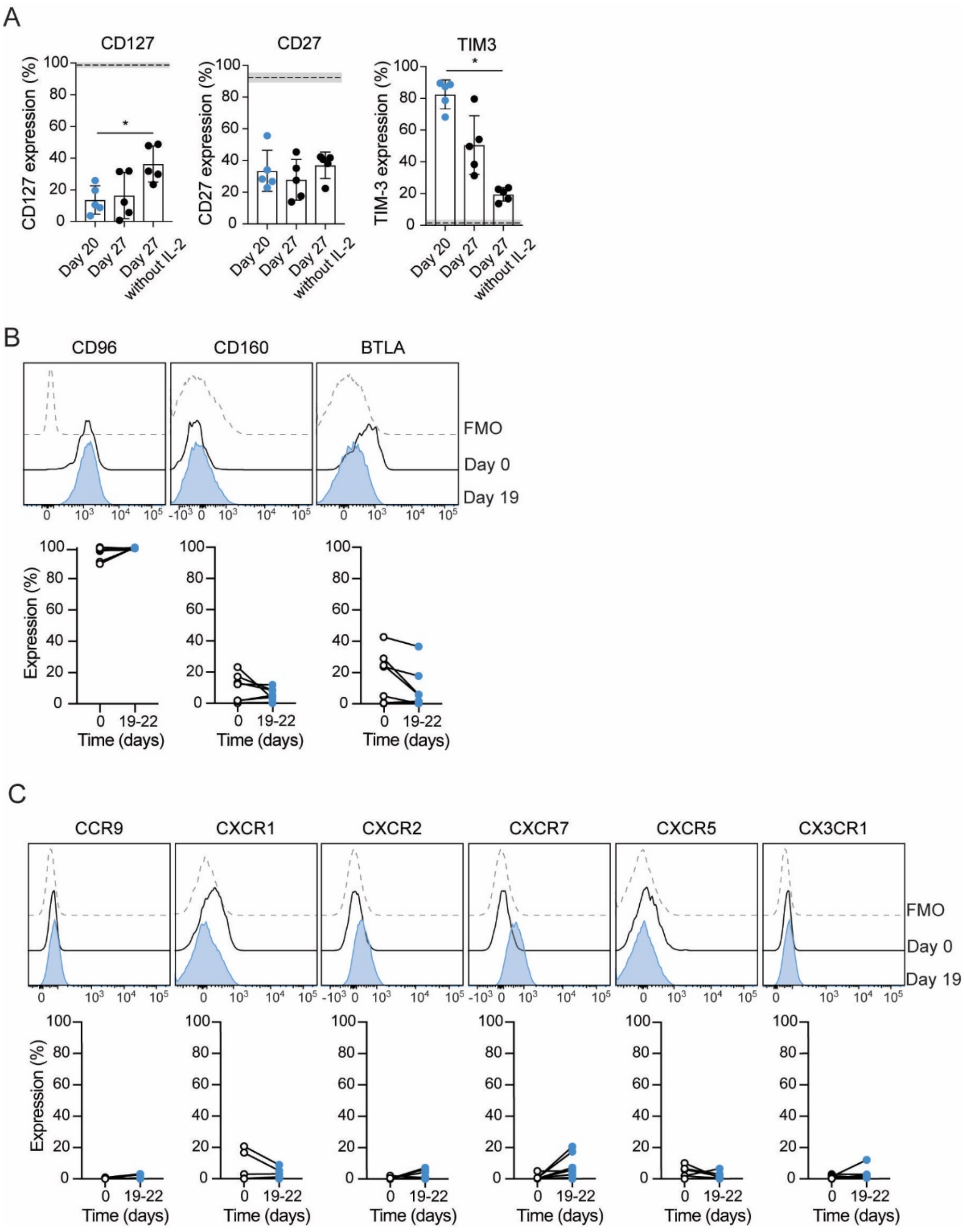

Supplemental Figure 3. Phenotypic comparison of MAIT cells before and after expansion. (A) Monitoring of CD127, CD27 and TIM3 expression on expanded MAIT cells over time. After 3 weeks of expansion, MAIT cells were kept in culture for 7 additional days in presence of 50 ng/mL IL-2 or without cytokine. Representative and average expression of the indicated (B) inhibitory receptors, and (C) chemokine receptors, on MAIT cells before (day 0) and after (day 19 to 22) expansion culture. In (A) the Friedman's test followed by the post-hoc Dunn's test was used for pair-wise non-parametric multiple comparison within each marker analysed. In (B, C) the Wilcoxon's signed rank test was used for statistical analysis of paired data. *$p < 0.05$.

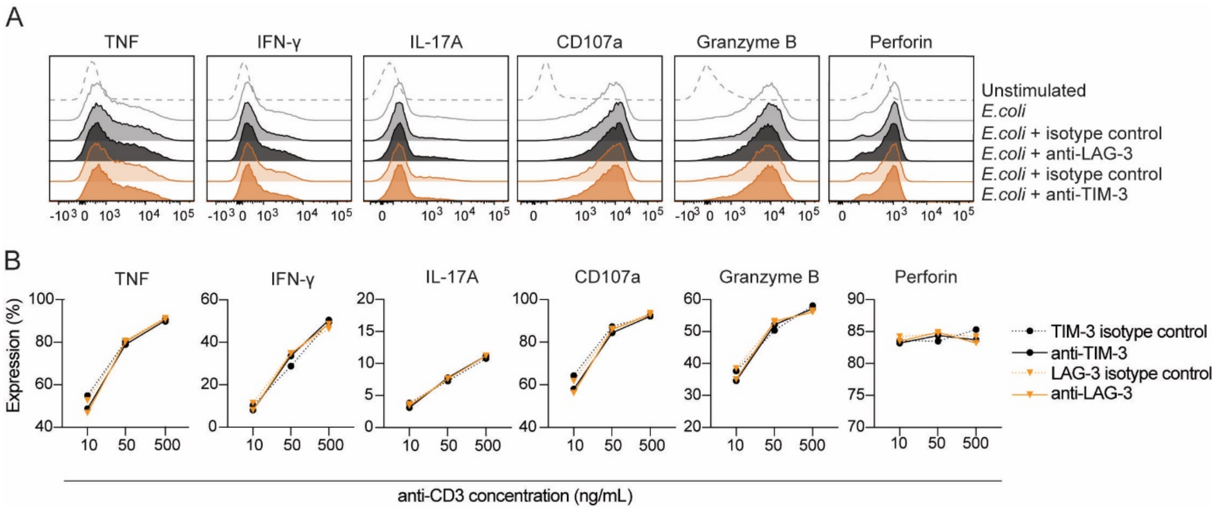

Supplemental Figure 4. Blockade and triggering of LAG-3 and TIM-3 receptors on expansion cultured MAIT cells. (A) Representative cytokine expression in expanded MAIT cells upon co-culture with *E. coli*-fed THP-1 cells for 24 h in presence of 10 µg/mL of anti-TIM-3 and anti-LAG-3 blocking antibodies or their respective isotype controls. (B) Percentages of expression of TNF, IFNγ, IL-17A, CD107a, granzyme B and perforin following stimulation for 6 h with P815 cells pre-coated with the indicated concentrations of anti-CD3 antibody in the presence of 10 µg/mL of the anti-TIM-3 and anti-LAG-3 blocking antibodies or their respective isotype controls. (A, B) Data from one representative donor out of four is shown.

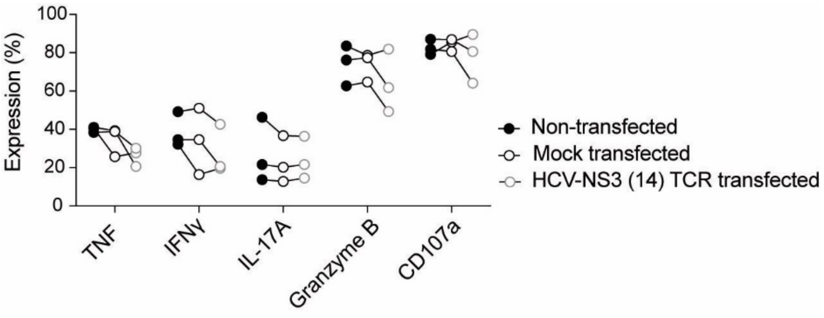

Supplemental Figure 5. Similar functional profile of MAIT cells before and after TCR transfection in response to bacterial stimulation. Percentages of expression of TNF, IFNγ, IL-17A, CD107a, and granzyme B in non-modified expanded MAIT cells, mock transfected or H4 HCV TCR transfected. MAIT cells were stimulated for 24 h with *E. coli*-fed THP-1 (n=3). The Friedman's test followed by the post-hoc Dunn's test were used for paired-wise non-parametric multiple comparison within each marker analysed.

Supplemental table 1. Estimated number of expanded MAIT cells obtained from 50 mL of buffy coat from healthy blood donors.

Donors	Number of PBMC (x10^6)[A]	% MAIT cells	Estimated number of MAIT cells (x10^6)[B]	Expansion fold[C]	Expected number of expanded MAIT cells (x10^6)[D]
1	750	0.90	6.75	140	945
2	870	0.63	5.48	179	981
3	660	2.12	14.00	129	1806
4	1000	0.70	7.00	152	1064
5	450	0.87	3.90	434	1693
6	500	2.50	12.50	315	3938
7	600	1.67	10.00	271	2710
8	400	2.16	8.65	389	3364
9	400	0.81	3.24	345	1119
10	828	0.97	8.00	221	1768
**Mean±SD**	**645.8±211**	**1.33±0.7**	**7.95±3.5**	**258±110**	**1939±1058**

[A]isolated from 50 mL healthy blood donor buffy coat

[B]estimation of the number of MAIT cells that could be isolated from 50 mL healthy blood donor buffy coat

[C]the expansion fold was calculated for each donor from the expansion of a portion of the isolated MAIT cells.

[D]if all the MAIT cells isolated from 50 mL healthy blood donor buffy coat were used for expansion.
